# Targeted Therapy for Acute Autoimmune Myocarditis with Nano-Sized Liposomal FK506 in Rats

**DOI:** 10.1371/journal.pone.0160944

**Published:** 2016-08-08

**Authors:** Keiji Okuda, Hai Ying Fu, Takashi Matsuzaki, Ryo Araki, Shota Tsuchida, Punniyakoti V. Thanikachalam, Tatsuya Fukuta, Tomohiro Asai, Masaki Yamato, Shoji Sanada, Hiroshi Asanuma, Yoshihiro Asano, Masanori Asakura, Haruo Hanawa, Hiroyuki Hao, Naoto Oku, Seiji Takashima, Masafumi Kitakaze, Yasushi Sakata, Tetsuo Minamino

**Affiliations:** 1 Department of Cardiovascular Medicine, Osaka University Graduate School of Medicine, Suita, Osaka, Japan; 2 Department of Pharmaceutical Chemistry, International Medical University, Kuala Lumpur, Malaysia; 3 Department of Medical Biochemistry, University of Shizuoka Graduate School of Pharmaceutical Sciences, Shizuoka, Japan; 4 Department of Internal Medicine, Meiji University of Integrative Medicine, Nantan, Kyoto, Japan; 5 Clinical Research and Development, National Cerebral and Cardiovascular Center Research Institute, Suita, Osaka, Japan; 6 Department of Cardiovascular Biology and Medicine, Niigata University Graduate School of Medical and Dental Sciences, Niigata, Japan; 7 Department of Pathology, Nihon University School of Medicine, Tokyo, Japan; 8 Department of Medical Biochemistry, Osaka University Graduate School of Medicine, Suita, Osaka, Japan; 9 Department of Cardiorenal and Cerebrovascular Medicine, Faculty of Medicine, Kagawa University, Miki, Kagawa, Japan; University of Nebraska-Lincoln, UNITED STATES

## Abstract

Immunosuppressive agents are used for the treatment of immune-mediated myocarditis; however, the need to develop a more effective therapeutic approach remains. Nano-sized liposomes may accumulate in and selectively deliver drugs to an inflammatory lesion with enhanced vascular permeability. The aims of this study were to investigate the distribution of liposomal FK506, an immunosuppressive drug encapsulated within liposomes, and the drug’s effects on cardiac function in a rat experimental autoimmune myocarditis (EAM) model. We prepared polyethylene glycol-modified liposomal FK506 (mean diameter: 109.5 ± 4.4 nm). We induced EAM by immunization with porcine myosin and assessed the tissue distribution of the nano-sized beads and liposomal FK506 in this model. After liposomal or free FK506 was administered on days 14 and 17 after immunization, the cytokine expression in the rat hearts along with the histological findings and hemodynamic parameters were determined on day 21. Ex vivo fluorescent imaging revealed that intravenously administered fluorescent-labeled nano-sized beads had accumulated in myocarditic but not normal hearts on day 14 after immunization and thereafter. Compared to the administration of free FK506, FK506 levels were increased in both the plasma and hearts of EAM rats when liposomal FK506 was administered. The administration of liposomal FK506 markedly suppressed the expression of cytokines, such as interferon-γ and tumor necrosis factor-α, and reduced inflammation and fibrosis in the myocardium on day 21 compared to free FK506. The administration of liposomal FK506 also markedly ameliorated cardiac dysfunction on day 21 compared to free FK506. Nano-sized liposomes may be a promising drug delivery system for targeting myocarditic hearts with cardioprotective agents.

## Introduction

Myocarditis is defined as an inflammatory disease of the myocardium caused by viral or bacterial infection, drugs, or autoimmune diseases [1]. Histological analysis shows the inflammatory infiltration of leukocytes within the myocardium [1,2]. Cytokines produced by leukocytes, such as T-lymphocytes and macrophages, play a crucial role in the pathogenesis of myocardial damage in myocarditis [1,3]. In the acute phase of myocarditis, the cytokines released from the T helper-1 (Th1) and T helper-17 (Th17) lymphocyte subsets are elevated in the myocardium [4,5]. Although immunosuppressive agents are believed to improve the survival of patients with immune-mediated myocarditis [6,7], the prognosis of some immune-mediated myocarditis, such as giant cell myocarditis (GCM), remains poor, and a more effective therapy must be developed [1].

Liposomes are nano-sized particles that are widely used for drug delivery to target specific organs with enhanced vascular permeability due to inflammation [8,9,10]. We have demonstrated that the targeted delivery of drugs to the ischemic/reperfused myocardium with liposomes has a promising therapeutic implication for cardiovascular diseases [11,12]. Because enhanced vascular permeability may occur in myocarditic hearts due to severe inflammation, we hypothesized that encapsulating an immunosuppressive agent within liposomes would increase its therapeutic effects.

FK506 is an immunosuppressive agent that suppresses T-cell activation by inhibiting calcineurin and decreases the level of interleukin (IL)-2 that triggers the release of cytokines from Th1 and Th17 lymphocytes [13]. We thus encapsulated FK506 in liposomes and examined the targeted accumulation of liposomal FK506 in experimental autoimmune myocarditis (EAM) rat hearts [14,15]. Additionally, we compared the effects of liposomal FK506 and free FK506 on the expression of cytokines along with the histological findings in the heart and hemodynamic parameters in EAM rats. This EAM model is characterized by the infiltration of T-lymphocytes and the appearance of multinucleated giant cells in the myocardium, and has been used as a disease model for GCM [16].

## Materials and Methods

### Materials

To prepare the liposomes, 1,2-dipalmitoyl-sn-glycero- 3-phosphocholine (DPPC), cholesterol, and 1,2-distearoyl-sn-glycero-3-phosphoethanolamine -N-poly(ethylene glycol) 2000 (DSPE-PEG) were obtained from Nippon Fine Chemical Co. (Takasago, Hyogo, Japan). FK506 was provided by Astellas Pharmaceutical Co., Ltd. (Tokyo, Japan). [^3^H]-FK506 was obtained from American Radiolabeled Chemicals, Inc. (St. Louis, MO, USA). All other materials were obtained from Sigma-Aldrich (St. Louis, MO, USA).

### Preparation of liposomal FK506

The lipid composition of liposomal FK506 was DPPC and DSPE-PEG in a 20:1 molar ratio. Liposomal FK506 was prepared as previously reported [14]. The particle size and zeta potential of liposomal FK506 were characterized by a dynamic light scattering analysis (Zetasizer Nano ZS; Malvern, Worcestershire, UK). The analyses were performed 15 times per sample, and the results represented the analysis of 3 independent experiments.

### Preparation of rat EAM model

Porcine cardiac myosin was prepared as previously reported [15]. Male Lewis rats (7 weeks old and weighing 190–220 g; Japan SLC, Inc., Shizuoka, Japan) received a single immunization in the footpads with a myosin-adjuvant emulsion according to the procedure described previously [17].

All animal experiments were performed in compliance with the guidelines of the Institute of Experimental Animal Sciences of the Osaka University Graduate School of Medicine. All rats were allowed ad libitum access to standard rodent chow and water, and housed in plastic cages maintained in a facility with a temperature of 23 ± 1.5°C and a 12 hr light/dark cycle. The protocol was approved by the Animal Care and Use Committee of the Osaka University Graduate School of Medicine (Approval number: 25-117-002) and all efforts were made to minimize suffering. Rats were euthanized by intraperitoneal injection with an overdose of pentobarbital (100 mg/kg), after measurement of hemodynamic parameters or before excision of hearts and other organs. The rats were monitored every day during the experimental procedure. Rats with weight loss ≥20% of the initial weight were scheduled to be euthanized, but no rat in this study met the requirement for early euthanasia. There was no rat died prior to the experimental endpoint.

### Immunohistological analysis

The immunohistological analysis was performed as described previously [18]. Sections were used for hematoxylin and eosin (HE) and Masson’s trichrome (MTC) staining and were examined by microscopy (BZ-9000, KEYENCE, Tokyo, Japan). The area percentages of cell infiltration and of fibrosis were calculated using Image J (NIH Freeware) [19].

### Experimental protocols

#### A. Time-course changes in vascular permeability in myocarditic hearts

On day 0, the rats received a single immunization to induce myocarditis. We examined the time-course changes in the vascular permeability in the myocarditic hearts of the EAM model rats on days 0, 10, 14, 17 and 21 after immunization. The rats were anesthetized with intraperitoneal sodium pentobarbital (50 mg/kg) and butorphanol (2.5 mg/kg). The fluorescent dye-labeled beads used in this study was carboxylate-modified beads with the diameter of 100 nm. These beads were labeled with red fluorescent (580/605; absorption/emission wavelength in nm, FluoSpheres F8801, Invitrogen, Carlsbad, CA, USA). The beads were dissolved in saline (500 μL/rat, 20% vol/vol) and were intravenously administered to the rats. Five minutes later, ex vivo fluorescence images were obtained using an Olympus SZX12 stereoscopic microscope equipped with a DP71 digital camera (Olympus, Tokyo, Japan).

#### B. Tissue distribution of liposomal FK506

To examine the tissue distribution of FK506, EAM rats on day 17 after immunization were anesthetized and received a single administration of liposomal [^3^H]-FK506 or free [^3^H]-FK506 via the jugular vein. Each rat received 0.01 mg of FK506 in a mixture of nonisotopic and [^3^H]-labeled FK506 at a 30:1 ratio (74 kBq/500 μL/rat). Two hours after administration, the rats were sacrificed for the collection of blood, heart, lung, liver and kidney specimens.

#### C. Effects of free or liposomal FK506 on heart inflammation and hemodynamic parameters

To examine the effects of free FK506, the rats received a single administration of 3 doses of free FK506 (0.035, 0.17 or 0.35 mg/kg) via the tail vein on days 14 and 17 after immunization. To examine the effect of liposomal FK506, a corresponding dose of free or liposomal FK506 (0.035 mg/kg and 0.17 mg/kg) was administered. On day 21 after immunization, the hearts were excised and the cytokine expressions in the myocarditic hearts were evaluated. In another series of experiments, the histological analysis and hemodynamic parameters were also analyzed on day 21 after immunization.

### Invasive measurement of hemodynamics

A 2-Fr Millar Mikro-tip catheter (SPR-320, Millar Instruments, Houston, TX, USA) was inserted via the right carotid artery and carefully introduced into the left ventricle. We obtained the hemodynamic parameters, such as heart rate (HR), left ventricular systolic pressure (LVSP), left ventricular end-diastolic pressure (LVEDP), and the maximum and minimum rates of left ventricular pressure (max dP/dt and min dP/dt). These parameters were analyzed using an application program Blood Pressure Module (LabChart, ADInstruments, Castle Hill, Australia).

### Measurement of [^3^H] radioactivity

The plasma and organ samples were obtained and the radioactivity was measured as previously reported [11]. The results were expressed as a percentage of the administered dose per 1 ml of plasma or 1 g of wet tissue weight.

### Real-time quantitative polymerase chain reaction

We obtained samples after the drug treatment and prepared them according to the Omniscript Reverse Transcription Handbook (QIAGEN Inc., Hilden, Germany). The rat primers used for the quantification of cytokines, including interferon-γ (IFN-γ), interleukin-17 (IL-17), tumor necrosis factor-α (TNF-α), interleukin-1β (IL-1β), interleukin-10 (IL-10), interleukin-4 (IL-4) and transforming growth factor-β (TGF-β), and glyceraldehyde-3-phosphate dehydrogenase (GAPDH) were all designed according to the manufacturer’s protocol (Applied Biosystems, Foster City, CA, USA; https://www.appliedbiosystems.com/). Real-time quantitative PCR was performed as described previously [18]. The primer sequences were as follows: IFN-γ, 5’-ctctctggctgttactgc-3’ (sense) and 5’-ccttttgccagttcctcc-3’ (antisense); IL-17, 5’-ttccacttcaccctggactc-3’ (sense) and 5’-tcccctcagcgttgacac-3’ (antisense); TNF-α, 5’-cccaacaaggaggagaag-3’ (sense) and 5’-tggtggtttgctacgacg-3’ (antisense); IL-1β, 5’-tgtgatgaaagacggcacac-3’ (sense) and 5’-cttcttctttgggtattgtttgg-3’ (antisense); IL-10, 5’-agtggagcaggtgaagaatga-3’ (sense) and 5’-tcatggccttgtagacacctt-3’ (antisense); IL-4, 5'-tgatgtacctccgtgcttga-3' (sense) and 5’-gtgagttcagaccgctgaca-3’ (antisense); TGF-β, 5’-gcaacacgtagaactctaccagaa-3’ (sense) and 5’-cagccactcaggcgtatca-3’ (antisense); GAPDH, 5’-tcaacggcacagtcaagg-3’ (sense) and 5’-cacgacatactcagcacc-3’ (antisense).

### Statistical analysis

One-way ANOVA followed by Tukey's statistics were used to determine the differences in the hemodynamic parameters, mRNA expression levels and histological findings among the groups. To compare the tissue distributions and hemodynamic parameters between the groups treated with free and liposomal FK506, unpaired t-tests were performed. The data are expressed as the mean ± SEM. In all analyses, P < 0.05 was considered to be statistically significant.

## Results

### Effects of free FK506 on hemodynamic parameters in the rat EAM model

**Fig 1:**
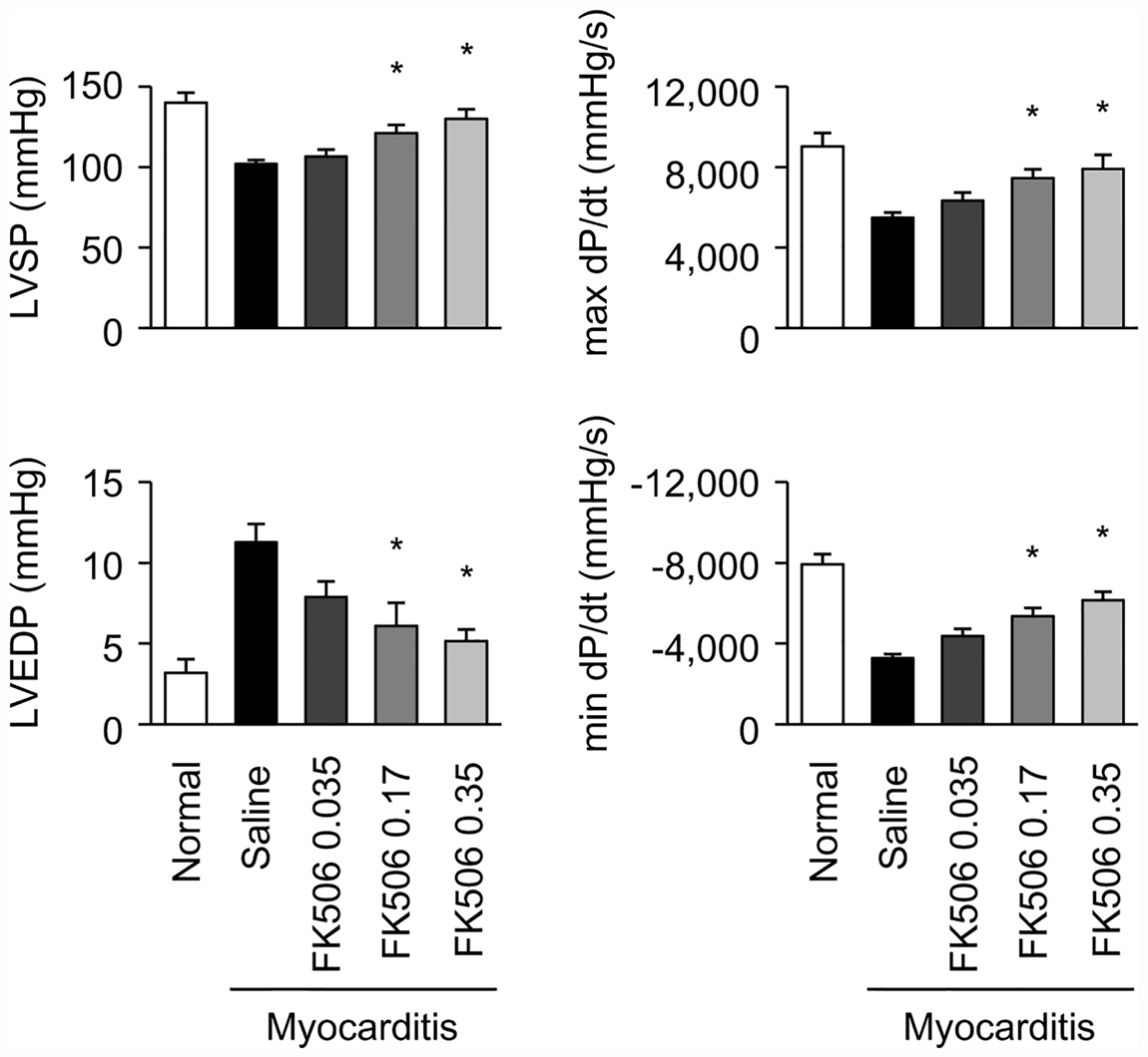
Effects of free FK506 on hemodynamic parameters in the rat EAM model. Quantitative data of hemodynamic parameters, including left ventricular systolic pressure (LVSP), left ventricular end-diastolic pressure (LVEDP), max dP/dt and minimum dP/dt, on day 21 after immunization. Drugs were intravenously administered on days 14 and 17 after immunization. Rats were divided into Normal, EAM with saline treatment and EAM with free FK506 (0.035, 0.17 or 0.35 mg/kg) treatment groups (N = 5–15 in each group). Data are expressed as the mean ± SEM. * P < 0.05 versus the saline treated group.

We tested the dose-dependent effects of free FK506 on the hemodynamic parameters on day 21 after immunization in the EAM rats. Free FK506 at the doses of 0.17 and 0.35 mg/kg, but not 0.035 mg/kg, improved the hemodynamic parameters, such as LVSP, LVEDP and dP/dt, on day 21 to the same extent (Fig 1). The HR in the normal rat group (417.1 ± 9.9 beats/min) did not differ from the other groups tested (Saline group: 368.4 ± 20.5 beats/min; FK506 0.035 mg/kg group: 364.5 ± 11.0 beats/min; FK506 0.17 mg/kg group: 389.2 ± 9.7 beats/min; FK506 0.35 mg/kg group: 390.8 ± 21.6 beats/min).

### Time-course changes in cardiac vascular permeability in the rat EAM model

**Fig 2:**
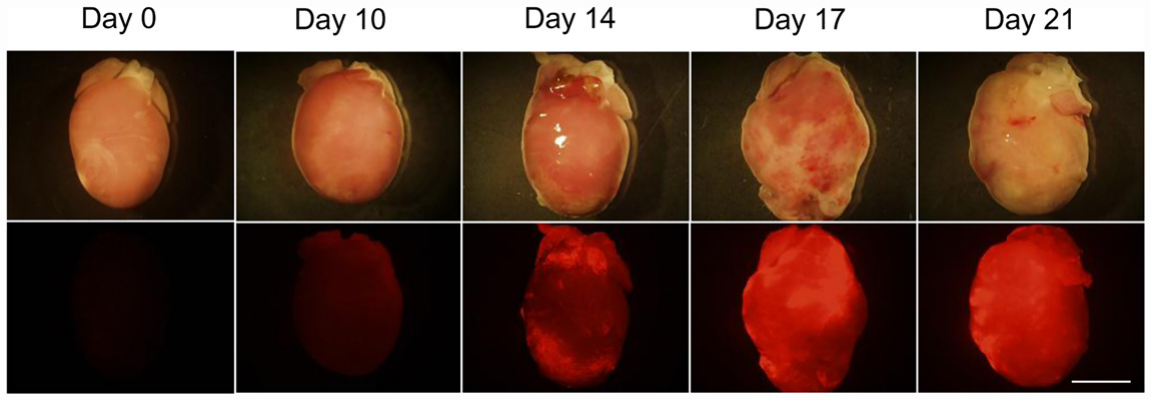
Time-course changes in vascular permeability in myocarditic hearts in the rat EAM model. The representative brightfield and fluorescent images of the myocarditic hearts of rats that received fluorescent-labeled nano-sized beads (100 nm) are shown in the upper and lower panels, respectively. Hearts were excised on days 0, 10, 14, 17 and 21 after immunization. In each time point, 2–3 rats were checked. Bar indicates 10 mm.

We assessed the time-course changes in the cardiac vascular permeability in the rat EAM model using nano-sized fluorescent beads (100 nm). An ex vivo fluorescent image analysis showed that an accumulation of nano-sized fluorescent beads was found in the hearts on day 14 after immunization and thereafter (Fig 2).

### Preparation of liposomal FK506 and tissue distribution of free and liposomal FK506 in the rat EAM model

We encapsulated FK506 into liposomes as previously reported [14]. The dynamic light scattering analysis showed that the particle size and zeta potential of the FK506 liposomes were 109.5±4.4 nm and -7.2±0.7 mV, respectively.

**Fig 3:**
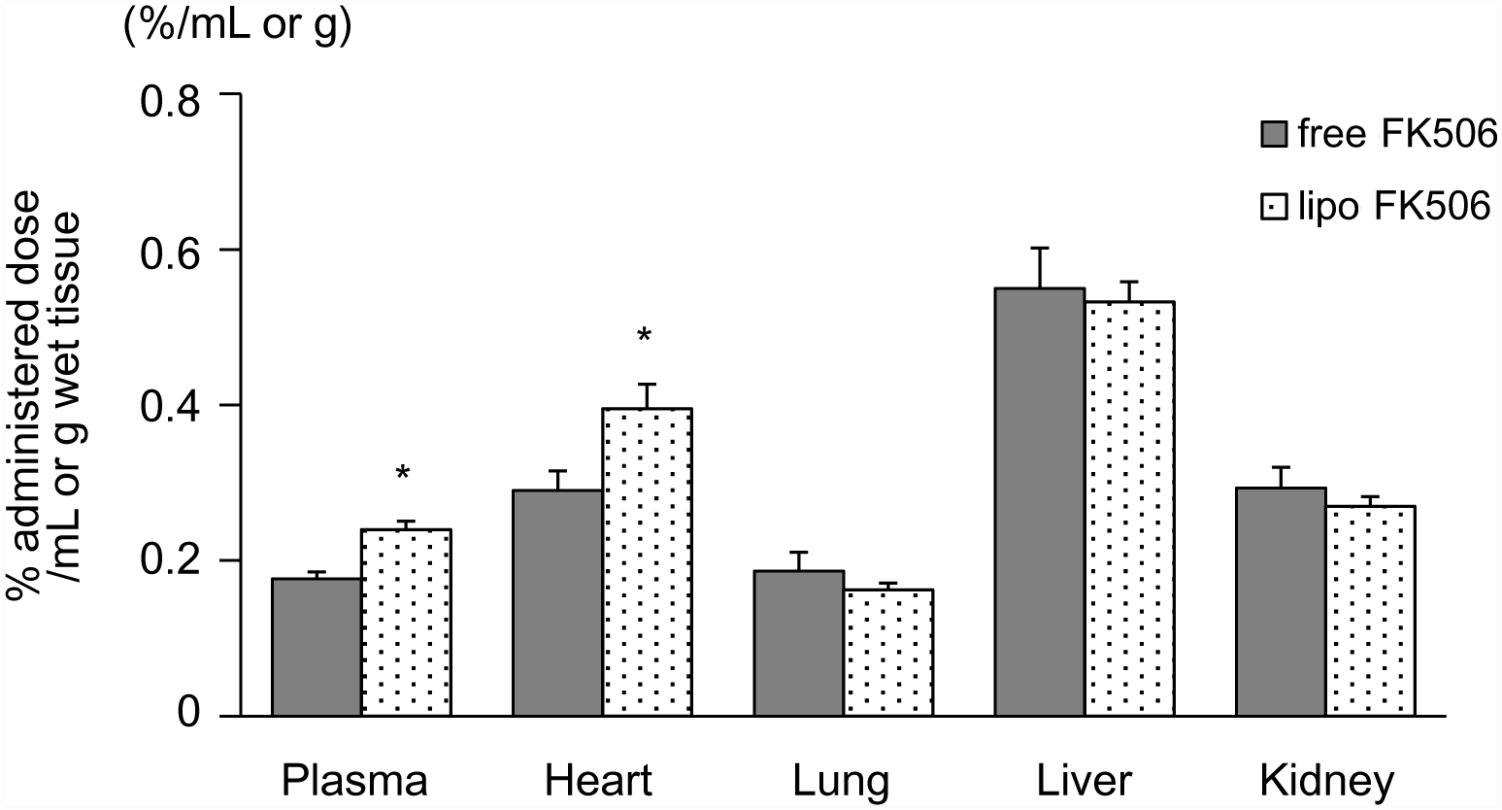
Tissue distribution of free and liposomal FK506 in the rat EAM model. Tissue distributions of [^3^H]-labeled FK506 in the rat EAM model. Radioisotope activity in tissues was measured 2 hours after the single intravenous administration of free or liposomal [^3^H]-labeled FK506 on day 17 after immunization (N = 4 in each group). Data are expressed as the mean ± SEM. * P < 0.05 versus the free FK506 treated group.

The plasma levels of [^3^H]-FK506 in the rats treated with liposomal FK506 were significantly higher than those treated with free FK506. The accumulation of radioisotopes in the heart, but not in the lung, liver, or kidney, was also significantly higher when rats received liposomal FK506 compared to free FK506 (Fig 3).

### Effects of free and liposomal FK506 on cytokine expression and histological findings in the rat EAM model

**Fig 4:**
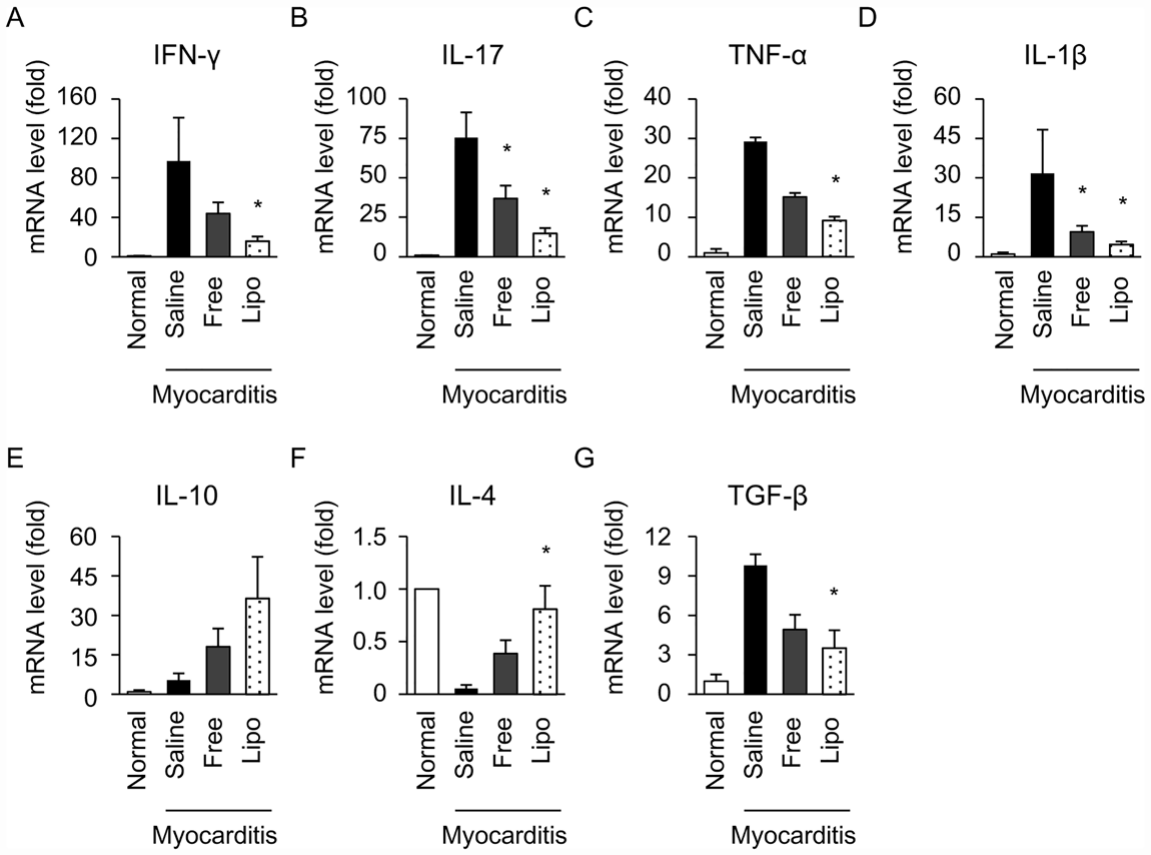
Effects of free and liposomal FK506 on inflammatory cytokines in the hearts of EAM rats. Cytokine expression on day 21 after immunization was evaluated using real-time quantitative PCR. Relative expression of IFN-γ (A), IL-17 (B), TNF-α (C), IL-1β (D), IL-10 (E), IL-4 (F) and TGF-β (G) were normalized to GAPDH (N = 4–9 in each group). Data are expressed as the mean ± SEM. * P < 0.05 versus the saline treated group.

Next, we examined the effects of free and liposomal FK506 on cytokine expression and histological findings in the rat EAM model. Because free FK506 at 0.17mg/kg and 0.35 mg/kg improved cardiac function of EAM rats to the same extent (Fig 1), we used an insufficient dose of free FK506 at 0.035 mg/kg to compare the therapeutic effects between free and liposomal FK506 on EAM. We examined the effects of free and liposomal FK506 on the mRNA expression of Th1 cytokine (IFN-γ), Th17 cytokine (IL-17) and proinflammatory cytokines (TNF-α and IL-1β) in rat myocarditic hearts on day 21 after immunization. Compared to the cytokine expression in myocarditic hearts treated with saline, liposomal FK506, but not free FK506, significantly decreased the expression of IFN-γ and TNF-α (Fig 4A and 4C). The expression of IL-17 and IL-1β were significantly decreased by both free and liposomal FK506 (Fig 4B and 4D). Because IL-10 was reported to have a therapeutic effect against autoimmune myocarditis [20], we investigated the expression of IL-10 in the hearts of EAM rats. IL-10 expression did not increase in EAM rats compared to normal rats on day 21. Furthermore, free or liposomal FK506 did not change the expression of IL-10 (Fig 4E).

Because Th2 cytokines have anti-inflammatory effects on this rat EAM model [21], we examined the expression of Th2 (IL-4) and found that liposomal, but not free FK506, significantly increased the expression of IL-4 (Fig 4F). Compared to saline, liposomal FK506, but not free FK506, significantly decreased the expression of TGF-β that induces fibrosis in this EAM model [22] (Fig 4G).

**Fig 5:**
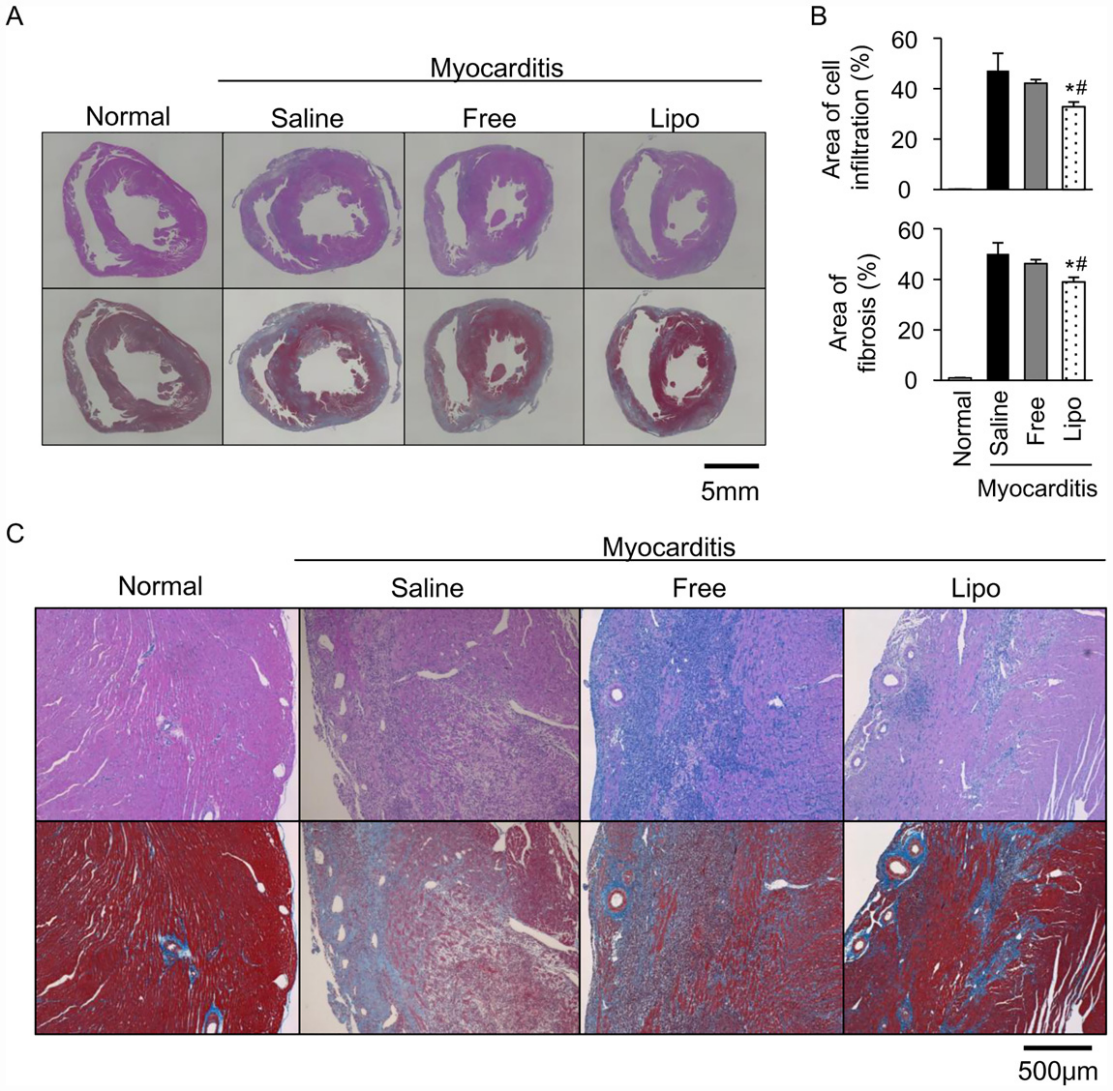
Effects of free and liposomal FK506 on histological findings in the myocarditic hearts. Representative transverse sections in ventricles stained with hematoxylin and eosin (upper panels) and Masson’s trichrome (lower panels) at low magnification (A) and high magnification (C). (B) Quantitative analysis of areas of cell infiltration and fibrosis (N = 4–12 in each group). Scale bars indicate 5mm and 500 μm in (A) and (C), respectively. Data are expressed as the mean ± SEM. * P < 0.05 versus the saline treated group. # P < 0.05 versus the free FK506 treated group.

We also examined the effects of free and liposomal FK506 on the histological findings at the dose of 0.035 mg/kg. Compared to saline, liposomal FK506, but not free FK506, significantly reduced the areas of cell infiltration and fibrosis in the myocarditic hearts. Moreover, liposomal FK506 significantly reduced the areas of cell infiltration and fibrosis than free FK506 in the myocarditic hearts (Fig 5A, 5B and 5C).

To investigate whether a higher dose of FK506 has more therapeutic effect on EAM than that of 0.035 mg/kg, we compared the histological findings between free and liposomal FK506 at the dose of 0.17 mg/kg. We found that both free and liposomal FK506 at the dose of 0.17 mg/kg reduced the areas of cell infiltration (free FK506: 28.52 ± 0.87%, n = 4, liposomal FK506: 26.96 ± 2.66%, n = 3 vs. saline: 42.39 ± 3.00%, n = 4) and fibrosis (free FK506: 42.28 ± 1.51%, n = 4, liposomal FK506: 38.83 ± 1.58%, n = 3 vs. saline: 51.00 ± 1.39%, n = 4) in the myocarditic hearts. These reductions to the saline treated group in the areas of cell infiltration (free FK506: 32.71%, liposomal FK506: 36.40%) and fibrosis (free FK506: 15.1%, liposomal FK506: 22.03%) by free and liposomal FK506 at 0.17 mg/kg were comparable with those by liposomal FK506 at 0.035 mg/kg (30.47% in the area of cell infiltration, 22.35% in the area of fibrosis). However, there was no significant difference in the anti-inflammatory effect between free and liposomal FK506 groups at the dose of 0.17mg/kg.

### Liposomal FK506 efficiently improved cardiac dysfunction in the rat EAM model

**Fig 6:**
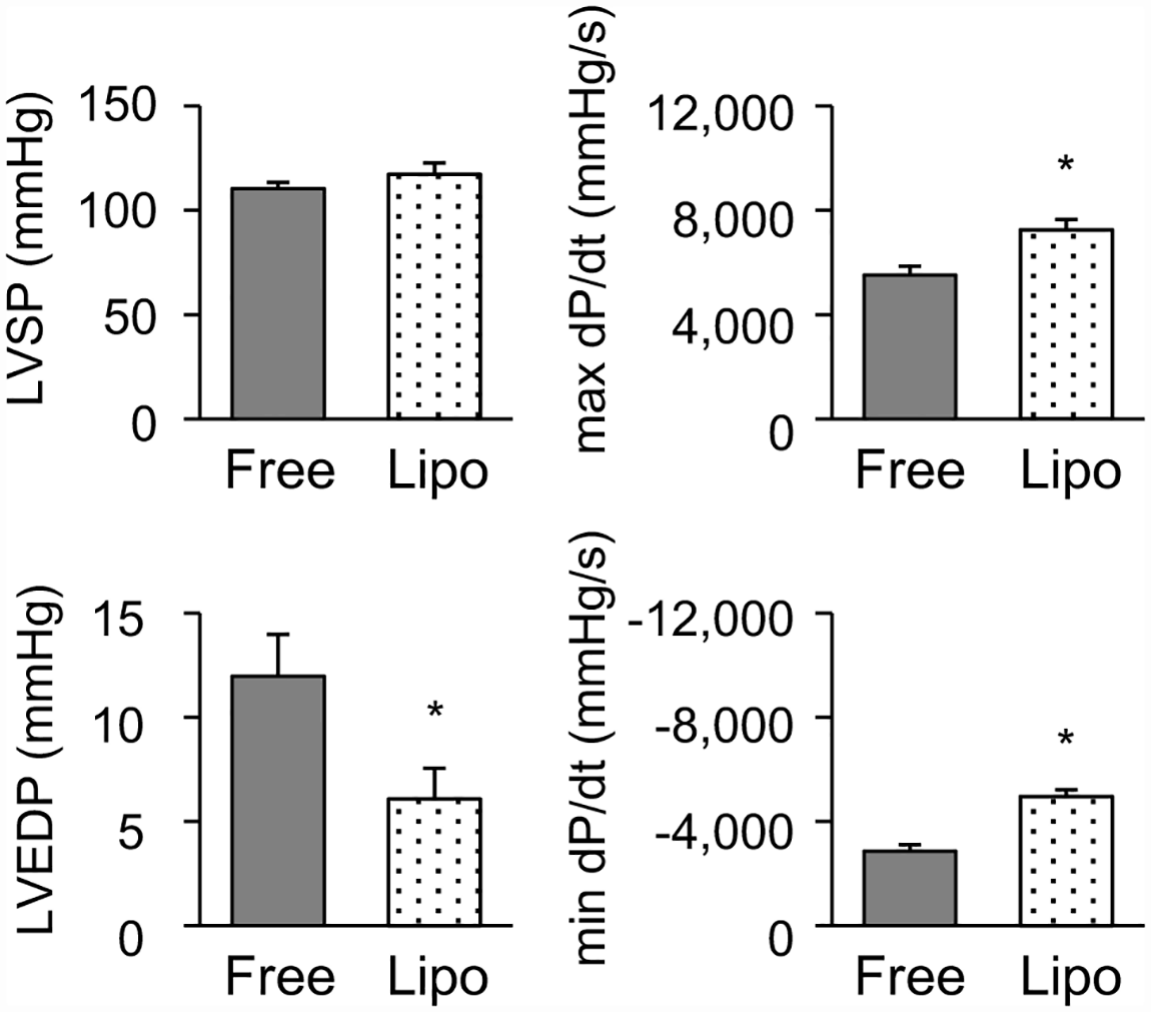
Effects of free and liposomal FK506 on hemodynamic parameters in the rat EAM model. Quantitative data of hemodynamic parameters including left ventricular systolic pressure (LVSP), left ventricular end-diastolic pressure (LVEDP), max dP/dt and minimum dP/dt. EAM rats were treated with free or liposomal FK506 (0.035 mg/kg) on days 14 and 17 after immunization (N = 6 and 9, respectively). Data are expressed as the mean ± SEM. * P < 0.05 versus the free FK506 treated group.

Next, we evaluated the effects of free and liposomal FK506 (0.035 mg/kg) on the hemodynamic parameters. Compared to free FK506, liposomal FK506 significantly improved the hemodynamic parameters, such as LVEDP and dP/dt, on day 21 after immunization (Fig 6).

We also compared the hemodynamic parameters between free and liposomal FK506 at the dose of 0.17 mg/kg. There was no significant difference in the therapeutic effect on hemodynamic parameters, including LVSP (free FK506: 102.7 ± 11.2 mmHg, n = 3 vs. liposomal FK506: 103.2 ± 7.0 mmHg, n = 4), LVEDP (free FK506: 6.3 ± 1.3 mmHg vs. liposomal FK506: 7.0 ± 0.4 mmHg), max dP/dt (free FK506: 5971.6 ± 1029.3 mmHg/s vs. liposomal FK506: 5681.7 ± 902.8 mmHg/s) and minimum dP/dt (free FK506: -5256.7 ± 821.6 mmHg vs. liposomal FK506: -5255.1 ± 701.5 mmHg/s) between free and liposomal FK506 groups.

## Discussion

In the present study, we demonstrated that nano-sized liposomes specifically accumulated in myocarditic hearts. Experiments with [^3^H]-labeled FK506 revealed that the plasma and heart tissue levels of FK506 in EAM rats were significantly higher when liposomal FK506 was intravenously administered compared to free FK506. Consistently, compared to free FK506 at the dose of 0.035 mg/kg, corresponding dose of liposomal FK506 suppressed cytokine expression in the hearts and improved the histological findings and cardiac function in EAM rats.

Immunosuppressive therapy has favorable effects in chronic virus-negative myocarditis, GCM and active myocarditis defined as autoimmune [2]. However, some patients with GCM still require mechanical circulatory support or a heart transplantation within 1 year [1,6]. Therefore, the development of a more effective immunosuppressive therapy in the acute phase of autoimmune myocarditis would meet a worldwide need.

Immunosuppressive therapy for immune-mediated myocarditis includes steroids alone, azathioprine and steroids, or cyclosporine A [1]. FK506 and cyclosporine A are calcineurin inhibitors, and they are used in clinical settings for patients received organ transplantation to prevent allograft rejection [13]. We chose FK506 due to its stronger bioactivity compared to cyclosporine A, which is suitable for encapsulation in liposomes [13]. Although the repetitive intramuscular administration of FK506 before or after the onset of inflammation has the potential to attenuate myocardial inflammation in the EAM model [23,24], the intravenous administration of FK506 after the onset of inflammation in the myocardium would be more applicable to a clinical setting. The initiation of myocardial inflammation occurs on days 11–14, and the cardiac dysfunction peaks on approximately day 21 after immunization [19]. We intravenously administered FK506 on days 14 and 17, which are periods after the onset of myocardial inflammation and before the peak of cardiac dysfunction. We chose 0.035 and 0.17 mg/kg of FK506, because 0.035 and 0.17 mg/kg/day are comparable with the insufficient and minimum effective dose in a rat allograft model, respectively [25,26]. In addition, we also examined the effect of a higher dose of 0.35mg/kg on hemodynamic parameters in EAM rats. Free FK506 at 0.17mg/kg and 0.35 mg/kg improved cardiac function of EAM rats to the same extent (Fig 1), indicating that free FK506 at 0.17 mg/kg attained a maximum therapeutic efficacy in rat EAM model. Therefore, we chose the submaximal dose of FK506 (0.035 mg/kg) to investigate whether liposomal formulation of FK506 can enhance the therapeutic efficacy of FK506.

Vascular permeability is often enhanced in tissues and organs in which inflammation is induced [8]. Nano-sized particles can pass through the vascular walls and accumulate in the interstitial tissues in which vascular permeability is enhanced [8]. Therefore, nano-sized particles such as liposomes are considered to be an ideal material to deliver drugs efficiently to inflamed tissues and organs such as those involved by cancer, infection and ischemia/reperfusion [8,9,10]. We have demonstrated that encapsulating drugs in liposomes augments their pharmaceutical effects in an ischemia/reperfusion rat model [11,12,27]. In the present study, along with the progression of myocardial inflammation, we found that nano-sized beads accumulated in the hearts of the EAM model rats (Fig 2). This finding indicates that nano-sized liposomes may be a promising drug delivery system for targeting myocarditic hearts with cardioprotective agents. Moffatt SD, et al. reported that a liposomal FK506 was efficacious in spite of low blood trough concentration of FK506, which brings the benefit for clinical organ transplantation [28]. They prepared the liposomal FK506 through 220 nm filters and obtained the liposomal FK506 which diameter was less than 220 nm. However, the peak and distribution of liposome diameter were not determined. On the other hand, we clearly characterized the liposomal FK506 which peak and distribution was 109.5±4.4 nm. Importantly, comparing to the liposome used in the previous study, our liposome was PEG-modified, which can potentially reduce uptake by phagocytic cells and enable liposome to remain in the blood circulation for long term [29]. Consistently, the FK506 level was significantly higher in the plasma in EAM rats treated with PEG-modified liposomal FK506, compared to that in EAM rats treated with free FK506 (Fig 3), suggesting that PEG-modification extended FK506 circulation time and improved its therapeutic effect against myocarditis. The accumulation of [^3^H]-FK506 in the whole heart was significantly increased (1.37-fold) in liposomal FK506 compared to free FK506. Because myocarditic hearts have focal inflammatory lesions wherein vascular permeability is enhanced, FK506 levels in the focal inflammatory lesions could be much higher for the liposomal FK506 group.

Cytokines produced by leukocytes, such as T-lymphocytes and macrophages, play a crucial role in the pathogenesis of myocardial damage in myocarditis [1,3]. To examine the immunosuppressive effects of liposomal FK506, we evaluated the mRNA expression of inflammatory cytokines in the hearts of EAM rats. Th1 and Th17 lymphocytes play an important role in the progression of immune-mediated myocarditis; therefore, we assessed the IFN-γ secreted from Th1 lymphocytes, IL-17 secreted from Th17 lymphocytes, and TNF-α and IL-1β secreted from macrophages [30,31,32]. Importantly, FK506 may suppress the function of T cells via calcineurin inhibition and may also directly suppress the production of a variety of T-cell-triggered cytokines such as IFN-γ, IL-17, TNF-α and IL-1β in several autoimmune diseases [13,33]. In this study, although we did not find statistically significant differences in each cytokine expression between free and liposomal FK506, liposomal FK506 significantly decreased the expression of 4 inflammatory cytokines (IFN-γ, IL-17, TNF-α, IL-1β) compared to saline, but free FK506 only decreased 2 of them (IL-17 and IL-1β) (Fig 4A, 4B, 4C and 4D). These findings suggested that liposomal FK506 may more efficiently suppress cytokine expression compared to free one. The combination of changes in cytokine expression by liposomal FK506 may synergistically contribute to the improvement of histological findings and hemodynamic changes in EAM rats.

We also examined the expression of anti-inflammatory cytokine, IL-10, on day 21 (Fig 4E). IL-10 expression did not increase in EAM rats compared to normal rats. Furthermore, free or liposomal FK506 did not change the expression of IL-10, indicating that FK506 improved histological findings and cardiac function independent of IL-10. Because IL-10 is reported to play an important role in the recovery phase (24–30 days after immunization of porcine myosin), the role of IL-10 is minimum in acute phase (day 21 after immunization) in the present study.

Moreover, Fuse K. et al. demonstrated that inflammatory Th1 cytokines (e.g. IFN-γ) suppress the expression of anti-inflammatory Th2 cytokines (e.g. IL-4) in the acute phase of EAM. In the present study, we also investigated the expression of IL-4 in the acute phase (day 21), because FK506 potentially suppresses the expression of Th1 cytokines, which may lead to increased expression of IL-4 in acute phase. As shown in Fig 4F, the expression of IL-4 was significantly increased in liposomal FK506 treated group, compared to that in saline treated group. These results indicated that the decrease of IFN-γ and the increase of IL-4 by liposomal FK506 in the hearts of EAM rats may contribute to the therapeutic effects of liposomal FK506 on EAM. In addition, we investigated the expression of TGF-β that was reported to induce cardiac fibrosis [22]. As shown in Fig 4G, TGF-β expression in the hearts of EAM rats was decreased by liposomal but not free FK506, comparing to that in the hearts of EAM rats treated with saline. These findings suggest that liposomal FK506 inhibited cardiac fibrosis via TGF-β signal pathway.

Next, we evaluated the therapeutic effects of liposomal FK506 on the histological findings in the hearts of EAM rats. In autoimmune myocarditis, cytokines induce a large amount of inflammatory cell infiltration at an early phase and fibrotic changes at a later phase, which leads to tissue atrophy and organ dysfunction [30]. Liposomal FK506 significantly reduced the areas of cell infiltration and fibrosis than free FK506 in the myocarditic hearts (Fig 5A, 5B and 5C).

Moreover, we evaluated the therapeutic effects of liposomal FK506 on the hemodynamic parameters. Compared to free FK506, liposomal FK506 significantly improved the hemodynamic parameters (Fig 6). Cytotoxic T-lymphocytes and macrophages, both of which are triggered by the activation of Th1 and/or Th17 lymphocytes, may directly damage cardiomyocytes and may also induce cardiomyocyte apoptosis [34]. Cytokines such as TNF-α and IL-1β themselves produce negative inotropic effects on cardiomyocytes [34,35]. We have demonstrated that liposomal FK506 significantly suppressed inflammatory cytokines, improved the histological findings, such as the reduced areas of cell infiltration and fibrosis, and improved the cardiac function.

We also challenged a higher dose of free or liposomal FK506 to investigate whether liposomal FK506 still has more therapeutic effect on EAM. Both free and liposomal FK506 treatment improved the histological findings to the same extent at the dose of 0.17 mg/kg, and the improvement compared to saline treated group was comparable with those treated by liposomal FK506 at 0.035 mg/kg. There was also no significant difference in hemodynamic parameters between free and liposomal FK506 treatment at the dose of 0.17 mg/kg. These results indicated that the therapeutic efficacy of free FK506 reached maximum level at the dose of 0.17mg/kg in this EAM rat model, and the increased accumulation of FK506 could not improve the therapeutic efficacy at the dose of 0.17 mg/kg. On the other hand, liposomal FK506 at the dose of 0.035 mg/kg was more effective than the free one in the treatment of EAM, which suggesting that the mechanisms of the improvement of therapeutic effects of liposomal formulation of FK506 at the submaximal dose (0.035 mg/kg) was due to the increased FK506 accumulation to the inflammatory tissue targeted by liposomes.

In conclusion, we observed that nano-sized particles specifically accumulated in myocarditic hearts and the level of FK506 was increased by liposomal FK506 administration compared to free FK506 administration. The increased accumulation of FK506 suppressed inflammatory cytokine expression, inflammatory cell infiltration and cardiac fibrosis, and ameliorated cardiac dysfunction, which suggested that liposomal FK506 treatment is an effective therapy for acute autoimmune myocarditis. These results indicated that the liposome is an effective method to enhance the therapeutic efficacy of FK506 in the acute phase of autoimmune myocarditis.
